# Association between indoor noise level at night and objective/subjective sleep quality in the older population: a cross-sectional study of the HEIJO-KYO cohort

**DOI:** 10.1093/sleep/zsac197

**Published:** 2023-01-27

**Authors:** Yuki Yamagami, Kenji Obayashi, Yoshiaki Tai, Keigo Saeki

**Affiliations:** Department of Epidemiology, Nara Medical University School of Medicine, Nara, Japan; Department of Epidemiology, Nara Medical University School of Medicine, Nara, Japan; Department of Epidemiology, Nara Medical University School of Medicine, Nara, Japan; Department of Epidemiology, Nara Medical University School of Medicine, Nara, Japan

**Keywords:** environmental noise, indoor noise, night noise, objective measures, actigraphy, sleep quality

## Abstract

**Study objectives:**

Noise exposure could be an important risk factor for low sleep quality; however, evidence on indoor noise in large-scale populations is limited. We evaluate the association between indoor noise at night and objective and subjective sleep quality in the older population.

**Methods:**

In this cross-sectional study of 1076 participants (≥60 years), we measured indoor noise at night (A-weighted equivalent noise from bedtime to rising time [*L*_Aeq_]) using a portable noise level meter set in bedrooms and sleep quality using actigraphy and a questionnaire for 2 nights. Using multivariable linear regression models, we examined the associations between indoor noise at night and objective and subjective sleep parameters independent of potential confounders such as age, body mass index, and sleep medication.

**Results:**

Increased indoor noise at night by 1 dB of *L*_Aeq_ was significantly associated with lower objective sleep quality, such as lower sleep efficiency (regression coefficient [*β*], −0.19%; 95% confidence interval [95% CI], −0.26 to −0.12; *p* < 0.001), longer log-transformed sleep onset latency (*β*, 0.02 log min; 95% CI 0.01 to 0.03; *p*< 0.001) and wake after sleep onset (*β*, 0.66 min; 95% CI 0.40 to 0.92; *p* < 0.001), and higher log-transformed fragmentation index (*β*, 0.01; 95% CI 0.008 to 0.017; *p* < 0.001). These results remained consistent in the analysis using noise-event rate (≥45 dB) as an independent variable.

**Conclusion:**

This study revealed the quantitative association between indoor noise at night and objective and subjective sleep quality in the older population. Reducing noise and improving sleep quality may prevent fatal diseases.

**Statement of Significance**

This cross-sectional study of 1076 participants examined the association between objective sleep measures using actigraphy and indoor noise at night using a portable noise level meter set in the bedroom. Poor objective sleep was significantly associated with increased indoor noise independent of potential confounding factors, such as age, BMI, snoring, current smoking, alcohol consumption, and sleep medication. These results remained consistent in the analysis using subjective sleep as measured by the participants’ self-reported questionnaires. Reducing noise and improving sleep quality may prevent fatal diseases.

## Introduction

Sleep disorders are common among older people and are associated with fatal diseases. The prevalence of sleep disorders among older people is from 12% to 45% [1, 2]. Systematic reviews indicated that poor sleep quality and decreased sleep duration are associated with increased all-cause mortality [3, 4], coronary heart disease, stroke [5], diabetes [6], and depression [7].

Over 10% of the population of European Union member countries (over 55 million people) are exposed to outdoor noise of >50 decibels (dB) at night (shown as *L*_night_, one of the night noise indicators) [8], although the World Health Organization (WHO) guidelines state that outdoor noise >40 dB at night is harmful to health [9]. Over 1 million disability-adjusted life years are lost in Western Europe due to traffic noise [10]. Meta-analyses with >590 000 and 110 000 participants have shown a dose–response relationship between noise and disease (coronary heart disease and hypertension) [11, 12]. Subanalysis of both studies at lower noise levels also retained a significant relationship.

Noise exposure could be an important risk factor for poor sleep quality. Several experimental studies suggested an association between noise and the probability of sleep stage change from deeper sleep stages to awake or stage 1 of non-REM sleep [13, 14]. A meta-analysis of observational studies has revealed a significant relationship between outdoor noise at night and subjective sleep disturbances [15]. However, previous studies had some limitations; for example, experimental studies do not reflect the effects of real-life noise [16], and many observational studies measured only outdoor noise regardless of only a fair correlation between indoor and outdoor noise (*r* = 0.48) [17]. The meta-analysis indicated that noise was significantly related to subjective sleep only when the questions specifically mentioned the noise [15].

Several observational studies with young participants reported the relationship between indoor noise and sleep measured with polysomnography [18–20]. However, the sample sizes were limited (40–94 participants), and the possibility of confounding could not be ruled out. External validity may be limited because participants living near traffic noise sources were only included. The participants were mainly young people, and the relationship between indoor noise and sleep in older people, who often experience noise-induced sleep disorders is unknown. This study aimed to investigate the association between indoor noise at night and objectively and subjectively measured sleep quality in the general older population.

## Methods

### Participants and the study protocol

A total of 1127 community-based older people (≥60 years) voluntarily participated between September and March 2010–2014 in a study entitled, “Housing environments and health investigation among Japanese older people in Nara, Kansai region: a prospective community-based cohort (HEIJO-KYO) study.” [21]

A total of 1053 participants completed measurements of indoor noise and actigraphic sleep for 2 consecutive days and 23 completed measurements for 1 of 2 days. One thousand seventy-six participants remained for analysis. All participants were provided written informed consent and the Nara Medical University ethics committee approved the study protocols.

### Measurement of indoor noise at night

A-weighted sound levels were measured at 1-min intervals for 2 consecutive nights using a portable noise level meter equipped with a windshield ball (SL-4023SD; Sato Shouji, Kanagawa, Japan; resolution, 0.1 dB), which was placed at the head of participants’ bed 30 cm above the floor. This device has a validated measurement range of 30–120 dB, although we have included raw data below 30 dB in the analysis.

We used the A-weighted equivalent continuous sound pressure level at night (*L*_Aeq_) as a parameter of noise exposure. We defined “night” as the period from bedtime to rising time according to a sleep diary recorded by the participants themselves. A-weighting indicates that sound pressure levels are weighted by frequency to mimic the sensitivity of the auditory system. *L*_Aeq_ was calculated using the following formula [22, 23]:


$$\begin{array}{l} L_{Aeq,\;\;\;T}\;\;\;\;\;=\;\;\;10\;\;\;log\frac{1}{t_{2}-t_{1}}\overset{t2}{\underset{t1}{\int }}10^{L\left(t\right)/10}dt \end{array}$$

*T* = *t*_*2*_ – *t*_*1*_, the observation interval.

*L*(*t*) = the instantaneous A-weighted sound level of noise at *t.*

*t*_*1*_ and *t*_*2*_ were defined as bedtime and rising time.

As another noise variable, a noise event was considered when the noise data were ≥45 dB on the noise data in each minute, and we calculated the noise-event rate per time in bed (hour). The thresholds of noise events (≥45 dB) are based on the WHO night noise guideline [9].

### Measurement of sleep parameters

Objective sleep quality was measured using actigraphy (Actiwatch2; Respironics Inc., Murrysville, PA) worn on the non-dominant wrist. The “wake” or “sleep” decision for each epoch was based on the following algorithm by the Actiware software (version 5.5, Respironics Inc) [24]. Activity counts of each 1-min epoch were calculated using the weighted moving average of the current epoch and the two preceding as well as the following two epochs as A = (0.04 × E_−2_) + (0.2 × E_−1_) + E + (0.2 × E_+1_) + (0.04 × E_+2_). En indicates the activity count 2 min before and after the epoch. If the weighted average activity A exceeded the predefined threshold of 40 counts/min, it was scored as awake and otherwise as asleep. The validity of this procedure in comparison with polysomnography has been confirmed previously (sensitivity, 0.93; specificity, 0.69) [24]. Sleep onset was defined as the first minute after a 10-min period of immobility of ≤4 counts/min. Sleep termination was determined as the last minute after a 10-min period of immobility [25]. To avoid overestimating the sleep/wake epoch, we used only epochs between self-reported bedtime and rising time for analysis.

Five sleep parameters were assessed using objectively measured data (sleep status and sleep onset and termination) and self-reported data (bedtime and rinsing time) as follows: (1) total sleep time (TST; total sleep epoch during the time in bed), (2) sleep efficiency (SE; the percentage of sleep epoch during the time in bed), (3) wake after sleep onset (WASO; total awake epoch between sleep onset and rising time), (4) sleep onset latency (SOL; the time between bedtime and sleep onset), and (5) fragmentation index (FI; the number of 1-min immobile epochs divided by the total number of immobile epochs during the time in bed) [26]. The averages for 2 consecutive days were used for analysis.

Subjective sleep quality was measured using the Pittsburgh Sleep Quality Index [27], a retrospective self-report questionnaire. Sleep quality over the previous month was determined using seven subscales that measured different components of sleep, namely sleep quality, sleep duration, SE, SOL, sleep disturbances, sleep medication use, and daytime dysfunction.

### Other measurements

Body mass index (BMI) was calculated as body weight (kg) divided by height (m^2^). Age, sex, current smoking status, drinking habit, education, economic status, and medication information were evaluated using a self-administered questionnaire. Nocturnal void frequency was assessed using a standardized urination diary. Nocturia was defined as two or more nocturnal voids at night, excluding the last void before bedtime and the first void after rising time. Snoring was determined by one of the Pittsburgh Sleep Quality Index questions. Physical activity was measured at 1-min intervals using an actigraphy worn on the non-dominant wrist for 48 hr. In the case of the participants for whom noise was measured for only one day, the physical activity data for the corresponding day were used.

### Statistical analyses

For analysis, the average values were used for the 1053 people with 2 days of noise and sleep data, whereas the remaining 23 people were analyzed using data from 1 day. Variables with normal distributions were reported as mean with standard deviation (SD), whereas variables with skewed distribution were reported as the median and interquartile range (IQR). SOL and FI values were natural logs transformed for analysis because of their skewed residual distributions of the model.

**Figure 1.**
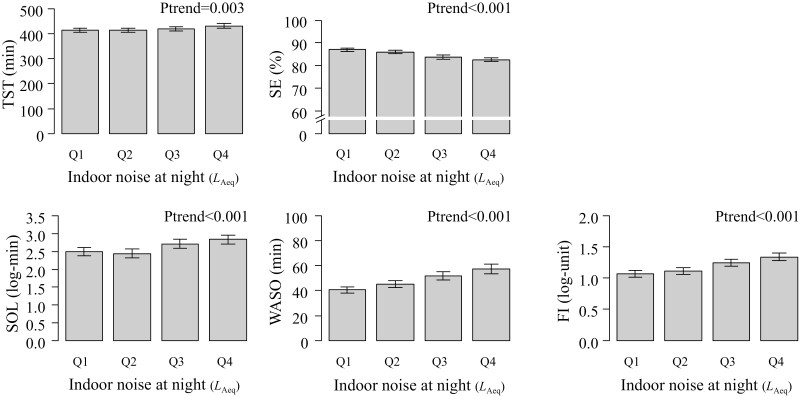
Unadjusted objective sleep parameters by quartile groups of indoor noise at night. Error bars indicate 95% confidence intervals of the means. The *P*-values were estimated using linear regression analysis. The median [range] indoor noise at night (*L*_Aeq_) in the quartile groups are Q1, 37.1 [31.0–39.36]; Q2, 41.7 [39.39–43.693]; Q3, 45.8 [43.695–48.128], and Q4, 51.2 [48.130–67.6], respectively.

**Figure 2.**
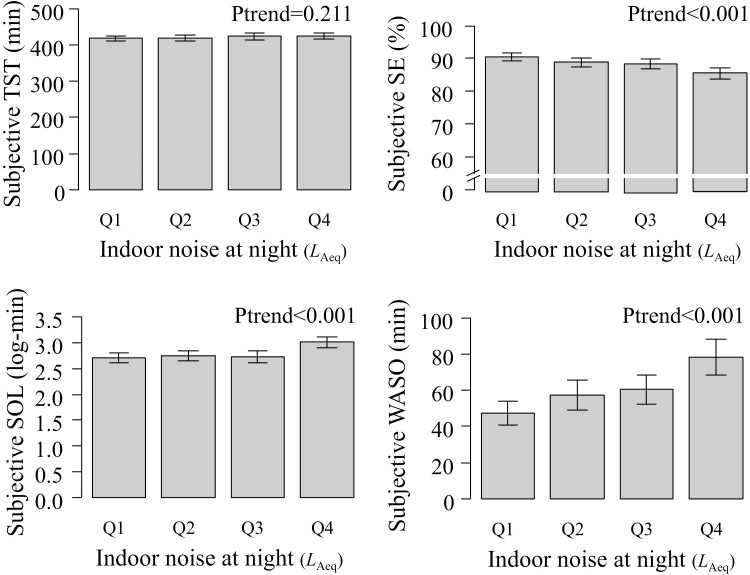
Unadjusted subjective sleep parameters by quartile groups of indoor noise at night. Error bars indicate 95% confidence intervals of the means. The *P*-values were estimated using linear regression analysis. The median [range] indoor noise at night (*L*_Aeq_) in the quartile groups are Q1, 37.1 [31.0–39.36]; Q2, 41.7 [39.39–43.693]; Q3, 45.8 [43.695–48.128], and Q4, 51.2 [48.130–67.6], respectively.

We divided the participants into quartile groups according to the intensity of noise exposure. Trends in the associations of the quartiles of indoor noise, basic and clinical parameters, and objective and subjective sleep parameters were evaluated. We used a linear regression model for continuous data of normal distribution, the Jonckheere–Terpstra trend test for continuous data of skewed distribution, and a logistic regression model for dichotomous data (Tables 1–3, Figures 1 and 2).

**Table 1.** Characteristics by quartile groups of indoor noise at night (*L*_Aeq_) (*n* = 1076)

Variables	All	Quartile groups of indoor noise at night *L*_Aeq_)				
		Q1	Q2	Q3	Q4	*p*_trend_
No. of participants	1076	269	269	269	269	
Indoor noise parameters						
Indoor noise at night (*L*_Aeq_), median [range], dB	44.1 [31.0–67.6]	37.1 [31.0–39.36]	41.7 [39.39–43.693]	45.8 [43.695–48.128]	51.2 [48.130–67.6]	
Noise-event rate (≥45 dB), median [range], counts/hour	3.2 [0.0–58.9]	0.8 [0.0–7.0]	2.2 [0.3–15.1]	5.1 [0.4–36.2]	13.6 [0.2–58.9]	
Basic and clinical parameters						
Age, mean (SD), years	71.8 (7.1)	70.5 (6.9)	71.0 (7.1)	72.3 (7.2)	73.6 (7.0)	<0.001
Male, number (%)	508 (47.2)	122 (45.3)	125 (46.5)	136 (50.6)	125 (46.5)	0.585
BMI, mean (SD), kg/m^2^	23.1 (3.1)	22.7 (2.9)	23.3 (3.3)	23.3 (3.0)	23.1 (3.0)	0.187
Snoring, number (%)	401 (37.3%)	91 (33.8%)	106 (39.4%)	104 (38.6%)	100 (37.2%)	0.173
Current smoker, number (%)	52 (4.8%)	11 (4.1%)	11 (4.1%)	16 (5.9%)	14 (5.2%)	0.374
Alcohol consumption (≥30 g/day), number (%)	155 (14.4%)	34 (12.6%)	35 (13.0%)	44 (16.4%)	42 (15.6%)	0.201
Education (≥13 years), number (%)	285 (26.4%)	90 (33.5%)	80 (29.7%)	69 (25.6%)	46 (17.1%)	<0.001
Household income (≥4 million JPY/year), number (%)	426 (42.8%)	107 (41.6%)	113 (45.2%)	111 (45.8%)	95 (38.8%)	0.576
Sleep medication, number (%)	117 (10.8%)	28 (10.4%)	24 (8.9%)	28 (10.4%)	37 (13.8%)	0.180
Antihypertensive medication, number (%)	481 (44.7%)	106 (39.4%)	115 (42.8%)	127 (47.2%)	133 (49.4%)	<0.001
Antidiabetic medication, number (%)	102 (9.5%)	16 (6.1%)	30 (11.2%)	23 (8.6%)	33 (12.3%)	<0.001
Nocturia (≥2 times/night), number (%)	308 (29.0%)	67 (25.2%)	71 (26.5%)	82 (30.8%)	88 (33.6%)	<0.001
Bedtime, mean (SD), clock time	22:34 (1:06)	22:44 (0:57)	22:45 (1:06)	22:27 (1:05)	22:18 (1:10)	<0.001
Rising time, mean (SD), clock time	6:46 (0:57)	6:40 (0:57)	6:46 (0:54)	6:44 (0:57)	6:52 (0:58)	0.021
Daytime physical activity, mean (SD), counts/min	297.8 (102.9)	286.9 (93.4)	307.1 (112.4)	297.3 (104.5)	300.0 (100.0)	0.297

Daytime physical activity was measured by actigraphy on the non-dominant wrist.

The *P*-values were estimated using linear regression analysis, logistic regression analysis, or Jonckheere–Terpstra trend test.

**Table 2.** Multivariable adjusted objective and subjective sleep parameters by quartile groups of indoor noise at night (*L*_Aeq_)

Variables			Quartile groups of indoor noise at night (*L*_Aeq_) [range (dB)]							
			Q1 [31.01–39.36]		Q2 [39.39–43.693]		Q2 [43.695–48.128]		Q4 [48.130–67.6]	*P*_trend_
No. of participants			269		269		269		269	
**Objective sleep parameters**										
TST, min	Adjusted mean* (95% CI)		418.4 (406.5 to 430.3)		422.6 (411.0 to 434.1)		415.2 (403.7 to 426.6)		421.0–409.6 to 432.4)	
	*P* value		reference		0.408		0.517		0.616	0.983
SE, %	Adjusted mean (95% CI)		84.8 (83.3 to 86.2)		84.2 (82.8 to 85.5)		82.3 (81.0 to 83.7)		81.4 (80.0 to 82.8)	
	*P* value		reference		0.324		< 0.001		< 0.001	<0.001
		Adjusted mean (95% CI)		2.49 (2.28 to 2.70		2.44 (2.23 to 2.65)		2.66 (2.46 to 2.86)		2,78 (2.58 to 2.98)
Log-transformed SOL, log min										
	*P* value		reference		0.572		0.057		0.001	<0.001
WASO, min	Adjusted mean (95% CI)		50.6 (45.2 to 55.9)		54.8 (49.5 to 60.0)		58.3 (53.1 to 63.5)		62.2 (57.0 to 67.3)	
	*P* value		reference		0.064		0.001		< 0.001	<0.001
Log-transformed FI	Adjusted mean (95% CI)		1.18 (1.08 to 1.27)		1.21 (1.11 to 1.30)		1.31 (1.22 to 1.40)		1.40 (1.31 to 1.49)	
	*P* value		reference		0.479		0.001		< 0.001	<0.001
**Subjective sleep parameters**										
TST, min	Adjusted mean (95% CI)		430.3 (416.4 to 444.2)		428.1 (414.6 to 441.6)	428.4 (415.0 to 441.7)			426.5 (413.2 to 439.9)	
	*P* value		reference		0.708		0.741		0.527	0.558
SE, %	Adjusted mean (95% CI)		89.9 (88.4 to 91.3)		88.1 (86.7 to 89.5)		88.5 (87.0 to 89.9)		86.6 (85.1 to 88.0)	
	*P* value		reference		0.083		0.179		0.002	0.012
Log-transformed SOL, log min		Adjusted mean (95% CI)		2.75 (2.58 to 2.92)		2.80 (2.64 to 2.97)		2.71 (2.55 to 2.88)		2.94 (2.77 to 3.10)
	*P* value		reference		0.461		0.614		0.010	0.041
WASO, min			48.1 (34.6 to 61.6)		60.0 (46.9 to 73.1)		56.1 (43.0 to 69.1)		68.1 (55.1 to 81.1)	
	*P* value		reference		0.037		0.166		0.001	0.002

^*^Adjusted for age, sex, BMI, snoring, current smoker, alcohol consumption, education, household income, sleep medication, antihypertensive medication, antidiabetic medication, nocturia, bedtime, and daytime physical activity.

**Table 3.** Multivariable adjusted objective and subjective sleep parameters by quartile groups of the noise-event rate at night over 45dB

Variables			Quartile groups of noise-event rate at night over 45dB (range [counts/hour])							
			Q1 [0.00–1.243]		Q2 [1.244–3.16]		Q3 [3.17–8.38]		Q4 [8.39–58.9]	*P*_trend_
No. of participants			269		269		269		269	
Objective sleep parameters										
TST, min	Adjusted mean* (95% CI)		424.3 (412.6 to 436.0)		414.8 (403.0 to 426.5)		416.7 (405.3–428.1)		421.4 (409.7–432.6)	
	*P* value		reference		0.056		0.133		0.532	0.636
SE, %	Adjusted mean (95% CI)		85.1 (83.6, 86.7)		83.8 (83.6, 86.6)		82.2 (81.2, 84.1)		81.5 (80.3, 83.2)	
	*P* value		reference		0.031		<0.001		<0.001	<0.001
Log-transformed SOL, log min		Adjusted mean (95% CI)		2.46 (2.25 to 2.67)		2.56 (2.35 to 2.77)		2.65 (2.45 to 2.86)		2.72 (2.51 to 2.92)
	*P* value		reference		0.261		0.027		0.004	0.002
WASO, min	Adjusted mean (95% CI)		50.3 (45.0 to 55.6)		53.5 (48.3 to 58.8)		58.6 (53.5 to 63.8)		63.5 (58.4 to 68.7)	
	*P* value		reference		0.150		<0.001		<0.001	<0.001
Log-transformed FI	Adjusted mean (95% CI)		1.17 (1.07 to 1.26)		1.23 (1.14 to 1.33)		1.31 (1.22 to 1.40)		1.39 (1.30 to 1.48)	
	*P* value		reference		0.091		<0.001		<0.001	<0.001
Subjective sleep parameters										
TST, min	Adjusted mean (95% CI)		430.3 (416.7 to 443.9)		428.0 (414.4 to 441.8)		434.5 (421.2 to 447.8)		420.0 (406.7 to 433.4)	
	*P* value		reference		0.704		0.472		0.085	0.198
SE, %	Adjusted mean (95% CI)		89.5 (88.1 to 91.0)		89.1 (87.6 to 90.5)		88.5 (87.1 to 89.9)		85.9 (84.4 to 87.3)	
	*P* value		reference		0.655		0.316		0.001	0.001
Log-transformed SOL, log min		Adjusted mean (95% CI)		2.71 (2.55 to 2.88)		2.72 (2.56 to 2.89)		2.84 (2.68 to 3.00)		2.91 (2.75 to 3.08)
	*P* value		reference		0.869		0.073		0.006	0.002
WASO, min	Adjusted mean (95% CI)		50.7 (37.5 to 64.0)		52.2 (38.9 to 65.6)		57.7 (44.7 to 70.6)		72.1 (59.1 to 85.1)	
	*P* value		reference		0.786		0.225		<0.001	<0.001

^*^Adjusted for age, sex, BMI, snoring, current smoker, alcohol consumption, education, household income, sleep medication, antihypertensive medication, antidiabetic medication, nocturia, bedtime, and daytime physical activity.

In multivariable linear regression models, after adjusting for potential confounding factors, objective and subjective sleep parameters were used as dependent variables, and indoor noise at night as an independent variable. We adjusted for age (per year), sex (male or female), BMI (kg/m^2^), snoring (yes or no), current smoker (yes or no), alcohol consumption (≥30g/day), an education level (≥13 years), household income (≥4 million JPY/year), sleep medication (yes or no) and antihypertensive medication (yes or no), antidiabetic medication (yes or no), nocturia (yes or no), bedtime (clock time), and daytime physical activity (counts/min). Comparisons of adjusted means for objective and subjective sleep parameters by quartiles of indoor noise parameters (*L*_Aeq_ and noise-event rate) were calculated using analysis of covariance (Tables 2 and 3). The missing values were two for BMI, 125 for snoring, 81 for household income, two for sleep medication, and 14 for nocturia. We imputed the mean for missing values of continuous value (BMI) and the overall proportions for the missing values of proportion (snoring, household income, and sleep medication). Furthermore, we conducted sensitivity analysis by converting data <30 dB into 20, 25, and 30 dB (Supplementary Tables S1–3).

Statistical analyses were performed using R version 4.1.0 for Windows [28], and a two-sided *P*-value of 0.05 was considered statistically significant.

## Results

The total measurement time for all participants was 538 640 min. Of these, 44 453 min (8.3%) were <30 dB. The percentage of data <30 dB for the individual was 0.0%–87.5%. The mean age of the 1076 participants was 71.8 (SD, 7.1) years, and 508 participants (47.2%) were male.

The average indoor noise at night (*L*_Aeq_), noise events at night (number/night), and the noise-event rate at night (counts/hour) of 1076 people were 44.1 [range, 31.0–67.6] dB, 25.5 [0.0–653.5], and 3.2 [0.0–58.9], respectively. Among the 1053 participants who completed 2 days of measurements, the night-to-night reproducibility of the indoor noise intensity was substantial (intraclass correlation coefficients, 0.64; 95% confidence interval [95%CI], 0.61 to 0.67). Higher indoor noise was significantly associated with older age (*p* < 0.001), a higher education level (*p* < 0.001), antihypertensive drug use (*p* < 0.001), antidiabetic drug use (*p* < 0.001), nocturia (*p* < 0.001), earlier bedtime (*p* < 0.001), and later rising time (*p* = 0.020; Table 1).

### Indoor noise and objective sleep parameters

In all participants, the mean TST was 420.6 (SD, 69.1) min, mean SE was 84.8 (SD, 7.4) %, median SOL was 18.5 (IQR, 9.5–35.5) min, mean WASO was 48.9 min (SD, 27.7), and median FI was 2.2 (IQR, 1.3–3.6). In univariable analysis, higher quartile groups of indoor noise (*L*_Aeq_) were significantly associated with the objectively measured sleep parameters, including longer TST (*p* for trend <0.003), lower SE (*p* for trend <0.001), longer log-transformed SOL (*p* for trend <0.001) and WASO (*p* for trend <0.001), and higher log-transformed FI (*p* for trend <0.001; Figure 1). Regarding the noise-event rate, higher quartile groups were significantly associated with longer TST (*p* for trend < 0.001), log-transformed SOL (*p* for trend < 0.001), and WASO (*p* for trend < 0.001), lower SE (*p* for trend < 0.001), and higher log-transformed FI (*p* for trend < 0.001).

In a multivariable analysis adjusted for potential confounders, these significant trends in quartile groups were consistent for SE (*p* for trend < 0.001), log-transformed SOL (*p* for trend < 0.001), WASO (*p* < 0.001), and log-transformed FI (*p* for trend < 0.001). The adjusted mean difference between the lowest (Q1) and highest quartile group of indoor noise (Q4) was significant for SE (−3.3%, 95% CI; −4.6 to −2.1), log-transformed SOL (0.29 log min, 95% CI; 0.11 to 0.47), WASO (11.6 min, 95% CI; 7.1 to 16.1), and log-transformed FI (0.22, 95% CI; 0.14 to 0.30; Table 2). Regarding the second-highest group (Q3), the adjusted mean difference between Q1 and Q3 was significant for SE (–2.4%, 95% CI; –3.6 to –1.2), WASO (7.7 min, 95% CI; 3.3 to 12.3), and log-transformed FI (0.13, 95% CI; 0.05 to 0.21; Table 2). Increased indoor noise by 1 dB of *L*_Aeq_ was significant associated with lower SE (*β*, –0.19%; 95% CI, –0.26 to –0.12; *p* <0.001), longer log-transformed SOL (*β*, 0.02 log min; 95% CI 0.01 to 0.03; *p* <0.001) and WASO (*β*, 0.66 min; 95% CI 0.40 to 0.92; *p* <0.001), and higher log-transformed FI (*β*, 0.01; 95% CI 0.008 to 0.017; *p* <0.001) independent of potential confounders. In the analysis using noise-event rate as an independent variable, these results remained consistent (Table 3).

The findings of the sensitivity analysis converting data below 30 dB into 20, 25, and 30 dB were also consistent with those of the original analysis (Supplementary Tables S1–S3).

### Indoor noise and subjective sleep parameters

The mean subjective TST, SE, and WASO were 422.4 (SD, 70.0) min, 88.2 (12.5) %, and 60.9 (69.8) min. The median SOL was 15.0 (IQR, 10.0–30.0) min. In univariable analysis, higher quartile groups of indoor noise were significantly associated with higher subjective SE (*p* for trend <0.001), longer log-transformed subjective SOL (*p* for trend <0.001), and longer subjective WASO (*P* for trend <0.001; Figure 2).

After adjusting for potential confounders, these significant trends were consistent for subjective SE (*p* for trend = 0.012), subjective log-transformed SOL (*p* for trend = 0.041), and subjective WASO (*p* for trend = 0.002). The adjusted mean difference between Q1 and Q4 was significant for SE (*β*, –3.3%; 95% CI, –5.3 to –1.2), log-transformed SOL (*β*, 0.19; 95% CI, 0.04 to 0.33), and WASO (*β*, 20.0 min; 95% CI, 8.6 to 31.3; Table 2). Increased indoor noise by 1 dB of *L*_Aeq_ was associated with lower SE (*β*, –0.15; 95% CI, –0.02 to –0.27; *p* = 0.01) and WASO (*β*, 1.01; 95% CI, 0.35 to 1.67; *p* = 0.003) independent of potential confounders. Regarding the noise-event rate, the significant trends between the quartile group of noise-event rate and subjectively measured sleep parameters were consistent (Table 3).

## Discussion

This large-scale study demonstrated a significant association between indoor noise at night and objectively measured sleep parameters, and we quantified the strength of the association. Increased indoor noise at night by 1 dB of *L*_Aeq_ was associated with a −0.19% decrease in SE, 0.66-min increase in WASO, 0.02 log min increase in log-transformed SOL, and 0.01 increase in log-transformed FI. These significant trends were consistent in the analysis using noise-event rate (≥45dB) as an independent variable. The sufficient number of participants in the present study (*n* = 1076) allowed us to adjust for potential confounders such as age, sex, BMI, the prevalence of snoring, smoking habits, alcohol consumption, and sleep medication.

The study had three strengths as follows: indoor noise measurements, assessment with sleep actigraphy, and a relatively large sample size. First, we precisely measured the amount of indoor noise exposure in the participants’ bedrooms. A previous observational study reported only a fair correlation between indoor and outdoor noise at the night (*r* = 0.48) [17]. Compared with indoor noise, the outdoor noise does not reflect the actual noise exposure of the participants, because of the variations in housing insulation and the location of windows. In some previous studies, indoor noise showed a stronger association with sleep quality than outdoor noise [17, 29]. Second, we objectively measured sleep quality using actigraphy independent of information bias to overestimate the influence of noise. Previous studies have indicated the possibility that the participants perceived that the noise had woken them up by hearing the noise after awakening, despite having spontaneous arousal [15]. Third, we included a larger number of participants than in the previous studies, which measured indoor noise and objective sleep quality. The sample size of the previous studies was relatively small (using polysomnography, *n* = 40–94, using actigraphy, *n* = 45) [18–20, 30, 31].

Compared with previous studies on young participants, our study showed that worse sleep quality was associated with noise exposure among older people. A non-randomized interventional study among 32 young participants (aged 19–28 years) showed that noise exposure (*L*_Aeq_, 39–50 dB) significantly reduced SE by 2% (93 vs. 91%) as compared with the control group (*L*_Aeq_, 32 dB) [14]. In another study among 72 participants (aged 18–71 years), compared with the control condition (*L*_Aeq_, 30.0 dB), participants with the noise condition (*L*_Aeq_, 36.9–43.3 dB) showed lower SE by 0.8% (88.9% vs. 88.1%) [31]. The reductions of SE by indoor noise in the previous studies were smaller than that found in the present study (the difference of SE: 3.3%) between Q1 (*L*_Aeq,_ 37.1 dB) and Q4 (*L*_Aeq_ 51.2 dB). These findings are consistent with the previous study that older participants were vulnerable to noise in sleep [32]. This study showed that higher indoor noise was significantly associated with older age. One possible mechanism is that snoring and movement during urination may have generated noise, given the increased prevalence of nocturia [33] and sleep-disordered breathing [34] with age. However, from the present results, it is unclear whether a direct relationship exists between age and indoor noise. The present study demonstrated a significant relationship between noise and sleep independent of age and age-related factors such as nocturia and snoring.

Consistent with previous epidemiological studies that investigated indoor noise, the present study showed that all participants were exposed to noise greater than the level recommended by WHO (*L*_Aeq_, 30 dB) [35]. A previous epidemiological study in France showed that the mean indoor noise level at night (*L*_Aeq_, from 10:00 pm to 6:00 am) in children’s bedrooms was 33.5 (SD, 4.6) dB [36]. In another observational study in the United States, the mean indoor noise level in the bedroom (*L*_Aeq_, from 10:00 pm to 8:00 am, 37.8 dB) of a group not exposed to traffic noise sources exceeded the WHO recommendation [37]. Complying with the WHO bedroom setting levels (30 dBA) in real life may be difficult given indoor noise levels presented in current and previous studies. Only a few studies have examined the relationship between actual indoor noise and sleep, and those who have done so have only included a small number of people. The present study is a large sample, but it is limited to older adults in a specific region of Japan. We assessed the reduction of sleep quality in Q2–Q4 compared with that in Q1 to estimate the influence of indoor noise. However, we might have underestimated the effect of noise because the participants in Q1 were exposed to noise beyond 33 dB, which could change sleep structure [19]. Further evidence is needed from studies measuring indoor noise in real-life situations in a large population with a wide range of ages and in various regions to determine the recommended level of preventing noise-related sleep disturbance.

Previous prospective cohort studies suggested the clinical implications of the noise-associated sleep disturbances examined in this study. The present study revealed that the adjusted mean SE of the highest indoor noise category (Q4) was 3.3% lower than the lowest (Q1; 81.4% vs. 84.7%). This difference in SE is larger than the difference (2.3%) with and without depressed mood (Geriatric Depression Scale score >5) between older people in a large community study (*n* = 3051; with: 81.2% vs. without: 83.5%) [35]. Reducing noise and improving sleep quality may prevent diabetes [6], dementia [38], cognitive decline [39], cardiovascular disease [5], and all-cause mortality [3].

The present study has several limitations. First, we were unable to determine the causal relationship between noise exposure and sleep. Body movements, household noises, and snoring may have all contributed to the noise generated by some participants with poor sleep quality. However, distinguishing environmental noises from participant-induced noises is difficult because our study recorded only noise levels and not noise sources or types. Because indoor sound directly reflects the participant’s daily life and privacy, it would be not easy to record the details of the sound. Second, our results may be distorted by the confounding effect of obstructive sleep apnea. Although we included the symptoms of snoring and nocturia as independent variables in the multivariable models, the residual confounding effect might have remained. Third, we could not distinguish the noise sources. Some studies indicated that different sources of noise sources might have different health effects, even at the same noise level [31, 40, 41]. Others reported similar effects of different noise sources [14]. Fourth, although the gold standard for objective sleep measurement is the polysomnography [42], used actigraphy to measure less invasively in a large population in this study. A reliability study of children and adolescents reported that actigraphy had acceptable validity compared to polysomnography on an epoch-by-epoch basis [24, 43, 44]. Fifth, participants’ hearing was not measured. A previous longitudinal study using a questionnaire found a significantly positive relationship between hearing loss and sleep duration [45]. However, to the best of our knowledge, the relationship between objective sleep quality and hearing remains unreported, and the mechanism by which hearing affects noise and sleep remains unclear. The possibility that the hearing may be a residual confounder cannot be ruled out, but further investigation is needed. Sixth, this study was conducted on older adults living in the Kansai region of Japan; hence, the results cannot be generalized. Further research on people of various ages and from various locations would be beneficial. Finally, selection bias was possible because the participants were not randomly sampled. However, the basic parameters such as BMI were consistent with the National Health and Nutrition Survey data conducted in 2010 [46]. The generalizability of our findings may be acceptable.

In conclusion, this study showed that indoor noise at night was significantly associated with objectively and subjectively measured sleep quality in the general older population.

## Supplementary Material

zsac197_suppl_Supplementary_Tables

## Data Availability

The datasets generated and analyzed during the current study are available from the corresponding author on reasonable request.
